# Translating the Behaviour Change Technique Taxonomy v.1 to other languages: the approach used in European Portuguese (BCTTv1-PT)

**DOI:** 10.12688/wellcomeopenres.20609.1

**Published:** 2024-03-01

**Authors:** Isa Brito Félix, Carolina C. Silva, Mara Pereira Guerreiro, Helga Rafael Henriques, Susan Michie, Maria Adriana Henriques, Marta M. Marques

**Affiliations:** 1 Egas Moniz Center for Interdisciplinary Research (CiiEM), Egas Moniz School of Health & Science, Monte Caparica, Portugal; 2 ADAPT Centre; School of Nursing and Midwifery, Trinity College Dublin, Dublin, Ireland; 3 Nursing Research, Innovation and Development Centre of Lisbon (CIDNUR), Nursing School of Lisbon, Lisbon, Portugal; 4 Centre for Behaviour Change, University College London, London, England, UK; 5 National School of Public Health, Comprehensive Health Research Centre, National School of Public Health, NOVA University of Lisbon, Lisbon, Portugal

**Keywords:** Behaviour change techniques, taxonomy, translation, BCTTv1, European Portuguese language

## Abstract

**Background:**

The Behaviour Change Techniques Taxonomy v1 (BCTTv1) is the most widely used classification of behaviour change techniques (BCTs), contributing to the accurate report and evaluation of behaviour change interventions and accumulation of evidence. This study reports a structured approach to adapt the BCTTv1 into European Portuguese (BCTTv1-PT).

**Methods:**

A collaborative and iterative approach was used. The translation process encompassed four phases: (1) independent forward translation by two native Portuguese speakers proficient in English, (2) forward translation reconciliation, (3) expert consultation by involving seven experts in behaviour change to collect feedback on the draft version of the taxonomy through a structured online form; and (4) feedback analysis and improvement of the BCTTv1-PT.

**Results:**

Independent forward translations and a reconciled version of the BCTTv1-PT were produced. All experts agreed with the groupings designation (100%). Recommendations were made to improve BCTs labels, definitions and/or examples in all groupings, except for *Feedback and monitoring*. Experts disagreed with the translated definitions in 40.9% of the BCTs (38/93), with examples in 21.5% (20/93) and with labels in 11.8% (11/93). Recommendations were made for all instances where there was disagreement (n = 69) and were enacted entirety, yielding the final version (BCTTv1-PT).

**Conclusions:**

Researchers, educators, students and health and other professionals will be able to standardise terminology and have a common language, contributing to the impact of the BCTTv1-PT. This study presents a systematic and rigorous approach for the adaptation of the BCTTv1 and similar taxonomies, which may guide others.

## Background

Behaviour change interventions (BCIs) – a set of activities, products, or services that aim to change specific behaviours – comprise multiple interacting components ( Michie *et al.*, 2021). Improving the design, implementation and evaluation of effective BCIs requires a clear description of their nature and content ( Michie *et al.*, 2021), a stance also endorsed by the UK Medical Research Council (MRC) guidance for developing and evaluating complex intervention ( Skivington *et al.*, 2021) and reporting guidelines ( Albrecht *et al.*, 2013; Hoffmann *et al.*, 2014).

The reasons for employing standardised language to describe and organise components of BCIs are multi-fold. It allows describing interventions as accurately as possible and therefore evaluating the effect of individual components, resorting, for example, to factorial designs. Further, standardised language enables the synthesis of published reports in systematic reviews and facilitates replication and implementation in practice.

The behaviour change technique taxonomy (BCTTv1) was developed by 400 experts from 12 countries ( Michie *et al.*, 2013). The taxonomy is composed of 93 clearly labelled and well-defined behaviour change techniques (BCTs) hierarchically organised in 16 groups, to improve ease of use. A BCT is an observable, replicable and irreducible component of an intervention designed to alter or redirect causal processes that regulate behaviour ( Michie *et al.*, 2013).

BCTTv1 applies to an extensive range of behaviour change interventions, in health and other areas, and has gained international acceptance as a tool for specifying their content ( Ismail *et al.*, 2020; McEvoy *et al.*, 2018; Murphy *et al.*, 2017; Ojo *et al.*, 2019; Schroé *et al.*, 2020). The availability of BCTTv1 in different languages is expected to promote acceptability in research, practice and education contexts in non-English speaking countries. An accurate translation is key to ensuring comparability between the original and the translated version of the taxonomy.

This study reports a structured approach to adapt the BCTTv1 into European Portuguese; by reflecting on this process, we offer potentially useful insights to others embarking on a similar endeavour.

## Methods

A collaborative and iterative approach was used to adapt the BCTTv1 into European Portuguese ( Douglas & Craig, 2007), to better respond to the complexity of the translation task, plus the range of expertise and the skills required.

**Figure 1.**
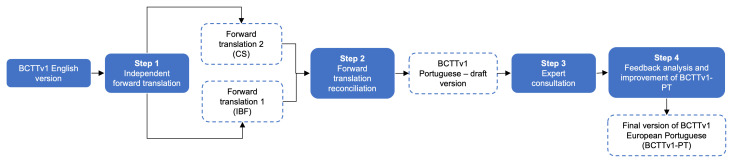
Translation process of BCTTv1 to European Portuguese.

The translation process encompassed four phases: (1) independent forward translation, (2) forward translation reconciliation, (3) expert consultation and (4) feedback analysis and improvement of the European Portuguese version of the BCTTv1 (BCTTv1-PT). Figure 1 outlines this process.

### Step 1: Independent forward translation

The forward translation from English to Portuguese was carried out by two independent translators: a nurse (IBF) and a behavioural scientist/health psychologist (CCS), both experienced in using the BCTTv1 for coding behaviour change intervention reports and in intervention development. Both translators were native Portuguese speakers proficient in English. General guidance was created based on agreed principles ( Open Science Framework, OSF1; Félix *et al.*, 2022). For instance, whenever possible, the BCTs label should be formulated as a noun and should be short.

### Step 2: Forward translation reconciliation

Using the two forward translations, a reconciled version of the BCTTv1-PT, designated as draft version, was produced by an independent translator (HRH, nurse), a native Portuguese speaker proficient in English. Additionally, specific concepts from psychology (e.g., cognitive structuring, shaping, coping planning) were discussed with another team member (MMM, behavioural scientist/health psychologist), to most accurately translate these concepts and definitions consistently across BCTs.

### Step 3: Expert consultation

A panel of seven experts in behaviour change was convened to collect feedback on the draft version of the taxonomy. These experts were native Portuguese speakers proficient in English, experienced in using BCTTv1 for coding intervention reports or for intervention development, and affiliated with academic and/or research institutions. Experts were identified through the research team network and invited by direct contact via email.

Feedback was collected through an on-line Google form ( OSF2; Félix *et al.*, 2022). To keep the workload manageable, each expert reviewed one to four groupings, depending on the number of BCTs in each grouping. Experts were asked about their agreement (Yes/No) with the translation of the (1) group label and (2) label, definition and example provided for each BCT in that grouping. In case of disagreement, experts were asked to provide recommendations for improvement using an open-ended question.

Written informed consent to participate in the consultation and to be acknowledged in the paper was obtained in the expert consultation step via the online survey.

### Step 4: Feedback analysis and improvement of BCTTv1-PT

Data originating from individual experts, in the form of excel spreadsheets, was merged into a single file, and subjected to descriptive statistics (frequency counts) for agreement on groupings designation, BCTs labels, and their definitions and examples. Recommendations were then discussed within the research team (IBF, CCS, MPG, HRH, MMM); changes were made in accordance, yielding the final BCTTv1-PT.

## Results

Independent forward translation originating from step 1 and the reconciled version of the BCTTv1-PT (step 2) are presented in the repository OSF3 ( Félix *et al.*, 2022) Next, we report findings from steps 3 and 4.

All experts agreed with the groupings designations (100%). Recommendations were made to improve BCTs **labels**, **definitions** and/or **examples** in all groupings, except for *Feedback and monitoring,* for which the reconciled version (draft) was endorsed by all experts. Experts disagreed with the translated definitions in 40.9% of the BCTs (38/93), with examples in 21.5% (20/93) and with labels in 11.8% (11/93). There were no missing answers. Recommendations were made for all instances where there was disagreement (n = 69).

Table 1 presents examples of the experts’ recommendations for **BCTs labels**. Recommendations were mainly focused on the use of more appropriate European Portuguese terms to enhance clarity.

**Table 1.** Example of experts’ recommendations for BCT labels.

BCTTv1 ( Michie *et al.*, 2013)		BCTTv1-PT draft	Recommendations
1.9	Commitment	Comprometimento	Compromisso
5.5	Anticipated regret	Antecipação do arrependimento	Visualização de arrependimento futuro
6.2	Social comparison	Comparação social com o desempenho do comportamento	Comparação social
9.3	Comparative imagining of future outcomes	Comparação de resultados futuros	Imaginação comparativa de resultados futuros
13.3	Incompatible beliefs	Convicções incompatíveis	Crenças incompatíveis
14.4	Reward approximation	Recompensa por aproximação	Recompensa por aproximações sucessivas

**Figure 2.**
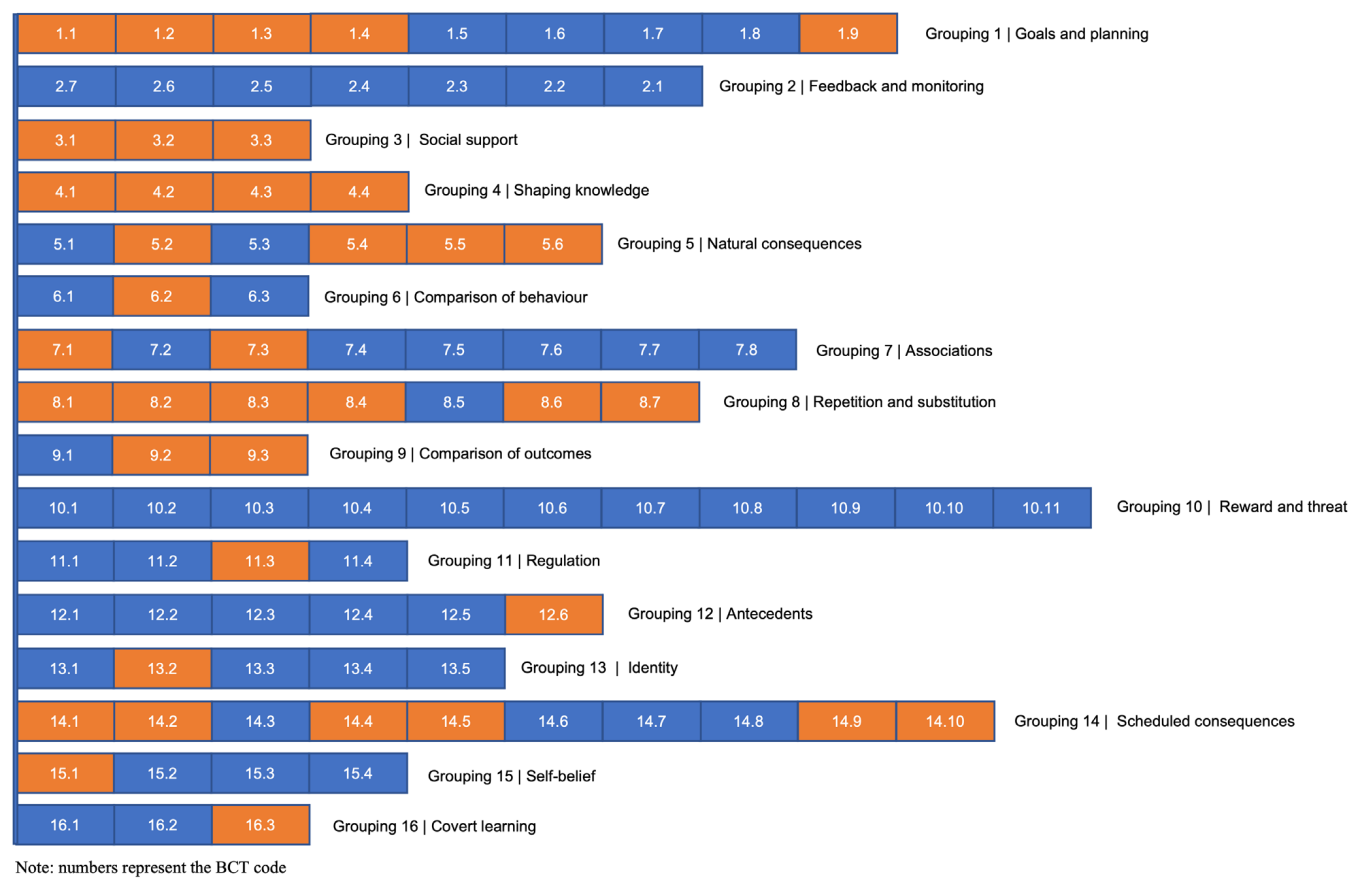
BCTs definitions that received recommendations for change (signalled in orange).

As depicted in Figure 2, no recommendations were made to the **definition of BCTs** included in grouping 2 ( *Feedback and monitoring)* and 10 *(Reward and threat)*. Examples of recommendations on this matter is presented in Table 2. In essence, these addressed (1) fidelity to BCT meaning; (2) consistency among definitions of different BCTs; and (3) terms regarded as more common in European Portuguese.

**Table 2.** Examples of experts’ recommendations for BCTs definitions.

BCTTv1 ( Michie *et al.*, 2013)			BCTTv1-PT draft		Recommendations	
	Label	Definition	Label	Definition	Label	Definition
1.4	Action planning	Prompt detailed planning of performance of the behaviour (must include at least one of context, frequency, duration and intensity). Context may be environmental (physical or social) or internal (physical, emotional or cognitive) (includes ‘Implementation Intentions’).	Planeamento da ação	**Incentivar** o planeamento detalhado da realização do comportamento (deve incluir pelo menos: contexto, frequência, duração ou intensidade). O contexto pode ser ambiental (físico ou social) ou interno (físico, emocional ou cognitivo) (inclui “Implementação de Intenções”)	Planeamento da ação	**Promover** o planeamento detalhado da realização do comportamento (deve incluir pelo menos: contexto, frequência, duração ou intensidade). O contexto pode ser ambiental (físico ou social) ou interno (físico, emocional ou cognitivo) (inclui “Implementação de Intenções”).
4.3	Re-attribution	Elicit perceived causes of behaviour and suggest alternative explanations ( *e.g., external or * *internal and stable or unstable*).	Reatribuições	Elicitar **perceções sobre** as causas do comportamento e sugerir explicações alternativas (e.g., externo ou interno e estável ou instável).	Reatribuições	Elicitar **as causas percebidas** do comportamento e sugerir explicações alternativas (e.g., externas ou internas; estáveis ou instáveis).
5.2	5.2 Salience of consequences	Use methods specifically designed to emphasise the consequences of performing the behaviour with the aim of making them more memorable (goes beyond informing about consequences).	Enfatizar as consequências	Utilizar métodos especificamente desenhados para enfatizar as consequências do desempenho do comportamento com o objetivo de as tornar memoráveis ( **vai mais longe que** informar sobre as consequências).	Enfatizar as consequências	Utilizar métodos especificamente desenhados para enfatizar as consequências do desempenho do comportamento com o objetivo de as tornar memoráveis ( **vai além de** informar sobre as consequências).
8.1	Behavioural practice/ rehearsal	Prompt practice or rehearsal of the performance of the behaviour one or more times in a context or at a time when the performance may not be necessary, in order to increase habit and skill.	Treino do comportamento	**Treinar o comportamento** uma ou mais vezes num contexto ou período de tempo em que pode não ser necessária a sua execução, visando o aumento do hábito e a habilidade.	Treino comportamental	**Incentivar o treino ou a prática do ** **comportamento** uma ou mais vezes num contexto ou período de tempo em que pode não ser necessária a sua execução, visando o aumento do hábito e a habilidade.
8.2	Behaviour substitution	Prompt substitution of the unwanted behaviour with a wanted or neutral behaviour.	Substituição do comportamento	**Substituir o comportamento** indesejado por um comportamento desejado ou neutro.	Substituição do comportamento	**Incentivar a substituição do ** **comportamento** indesejado por um comportamento desejado ou neutro.
8.7	Graded tasks	Set easy-to-perform tasks, making them increasingly difficult, but achievable, until behaviour is performed.	Tarefas progressivas	Definir tarefas fáceis de executar, tornando-as cada vez mais difíceis até que o comportamento seja desempenhado	Tarefas progressivas	Definir tarefas fáceis de executar, tornando-as cada vez mais difíceis, **mas sempre alcançáveis**, até que o comportamento alvo seja desempenhado.

Lastly, 20 **BCTs examples** received recommendation for change. For instance, “Advice to keep biscuits and snacks in a cupboard that is inconvenient to get to” is the example provided for BCT “Restructuring the physical environment” (12.1); biscuits were recommended to translate to *bolachas* as it is a more common term used Portuguese, instead of *biscoitos*. Overall, recommendations intended mainly to guarantee harmonization across BCTs wording (e.g., changing the infinitive form of verbs to non-infinitive forms) or to adapt the wording to the Portuguese context.

**Figure 3.**
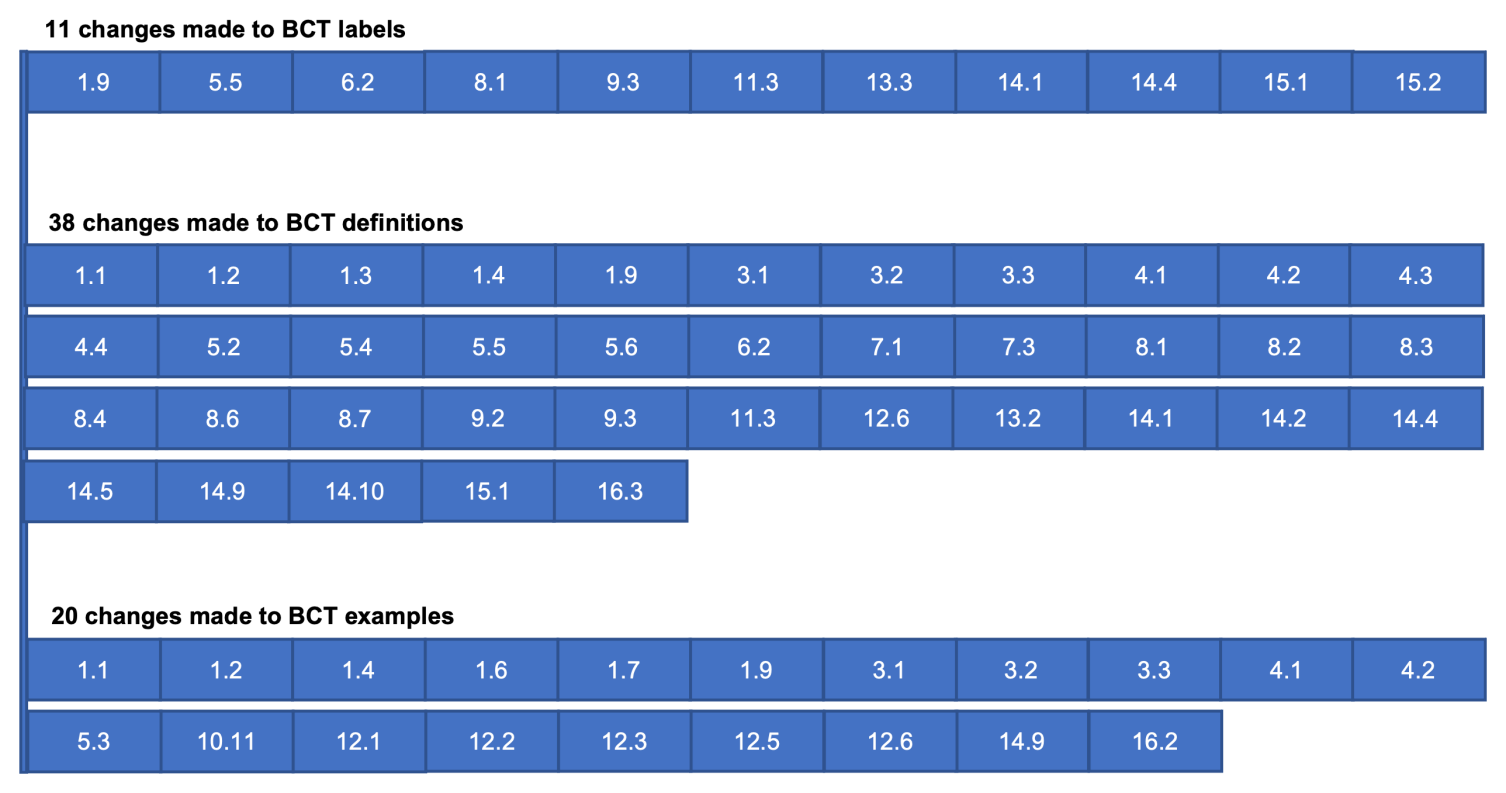
BCT labels, definitions and examples changed.

Experts’ recommendations were enacted in their entirety; Figure 3 details BCTs subjected to changes as a result of feedback analysis.

At this stage, typos and minor linguistic errors were identified and amended, yielding the final version (BCTTv1-PT) ( OSF4; Félix *et al.*, 2022).

**Figure 4.**
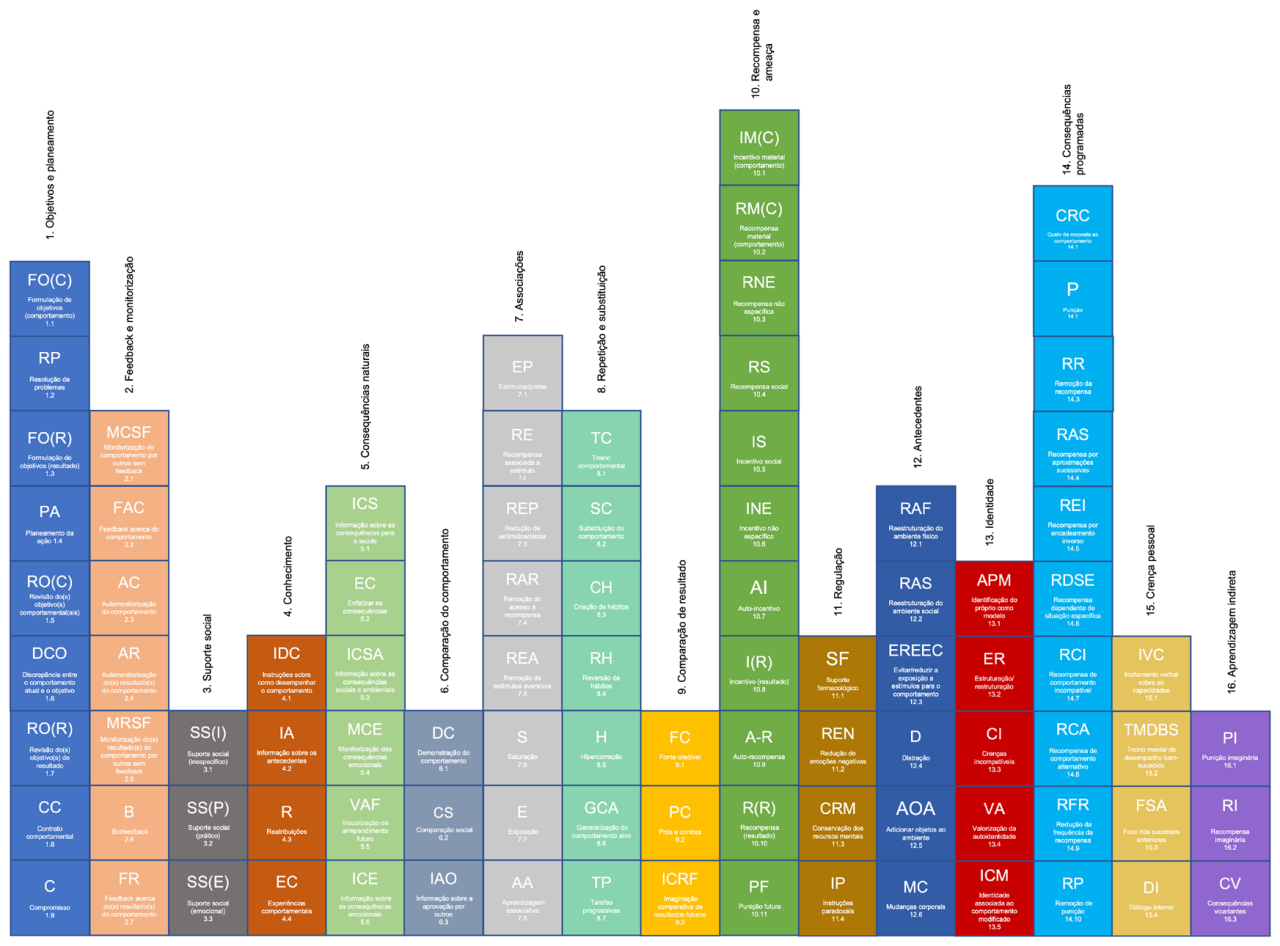
The “periodic table” of behaviour change techniques, version 1, European Portuguese (BCTTv1-PT).

To facilitate use in practice, a visual representation of BCTTv1-PT was developed ( Figure 4), based on the work of Armitage *et al.* (2021) and Michie *et al.* (2013). Although the “periodic” table of behaviour change techniques is not hierarchically structured, we believe that it can still be helpful to those seeking an overview of the taxonomy.

## Discussion

This paper describes a collaborative, interdisciplinary, systematic and iterative approach of adapting the original version of the BCTTv1 to European Portuguese (BCTTv1-PT) This laborious process encompassed a total of 69 changes from the reconciled version resulting from experts’ recommendations, either in the BCTs label, definition or example. These recommendations were made on a reconciled draft from two forward translations, which already encompassed substantive iteration. This illustrates the importance of considering a range of views to maximise rigour in translation.

Our work has important implications for research, education and practice, both in Portugal and other Portuguese-speaking countries, which altogether account for over 250 million speakers. Although differences exist among European Portuguese and Portuguese spoken in these countries, others have produced versions of instruments to be used in more than one country from this community (e.g. Salgado *et al.*, 2013 - Portugal and Brazil). At a bare minimum, BCTTv1-PT is expected to be useful as a starting point for other Portuguese-speaking countries, reducing considerably the burden of translation.

Interventions delivered by professionals in a national research context may benefit from using BCTTv1-PT to increase fidelity, as the original English version is prone to unintended deviations in meaning.

The translation of BCTTv1 to European Portuguese is expected to facilitate education and training, both at undergraduate and postgraduate levels, as a resource in learners’ native language may mitigate common domain specific difficulties when first studying this topic. Ultimately, this resource may contribute to overcoming barriers for delivering behaviour change interventions in practice, such as perception of lack of skills and lack of confidence ( Keyworth *et al.*, 2020).

Lastly, it is also envisaged that BCTTv1-PT will facilitate accurate recording of behaviour change interventions in practice, ensuring consistency between interventions. It may contribute to increased recognition of the breadth of professionals’ practice by unveiling unreported interventions.

### Strengths and limitations

One of the strong points of our work is the collaborative and iterative approach followed which contributes to a consolidated version. Methodological approaches to translation, adaptation and cross-cultural validation of research instruments or scales ( Sousa & Rojjanasrirat, 2011) were described in the literature. Typically, these approaches encompassed sequential procedures such as forward translation, synthesis of translated versions, backward translation (blind), pretest of synthesised translated version and psychometric testing ( Efstathiou, 2019; Sousa & Rojjanasrirat, 2011). These procedures are not entirely transferable to the adaptation of the BCTTv1 due to their distinct nature; for example, the taxonomy does not have clusters of correlated items. Further, the method used in this study can be replicable for the translation and adaptation of other classifications of behaviour change techniques (e.g., the Behaviour Change Techniques Ontology currently developed) and of other components of BCIs such as the mode of delivery of BCIs ( Corker *et al.*, 2023; Marques *et al.*, 2021).

Guidelines on translation and adaptation were not found in the literature. There are publications on the translation and cross-cultural adaptation of taxonomies. Haag and colleagues (2020) performed an online Delphi survey to translate the ABC taxonomy for medication adherence. A similar study was also conducted to translate this ABC taxonomy into Portuguese ( Bernardo *et al.*, 2023); the authors identified published taxonomy terms and definitions in Portuguese through a systematic review and then sought consensus using a Delphi method. The first step of this approach would be challenging to apply, and we would not find labels, definitions and examples for all BCTs in literature. Given the scarcity of resources, namely bilingual behavioural psychologists with a vast experience in working with BCTTv1, using a consensus method was considered a non-feasible method. A modification of the collaborative and iterative approach followed ( Douglas & Craig, 2007) was introduced to ensure the steps were more suitable to the translation of a taxonomy. Thus, this study presents a systematic and rigorous approach for the adaptation of the BCTTv1 and similar taxonomies, which may guide others.

Another strength is the involvement of an interdisciplinary team, in particular behavioural psychologists, which ensures the linguistic/literal equivalence and fidelity of the translation into European Portuguese.

A limitation of this study is that the final version of BCTTv1-PT has not been pilot tested. Potential ambiguities and discrepancies could be improved by providing intervention descriptions and asking participants to code the existing BCTs in Portuguese and English.

## Conclusions

A translated version of the BCTTv1 into European Portuguese is available for the first time (BCTTv1-PT). The translated taxonomy is thought to be used in research, education and practice settings. Researchers, educators, students and health and other professionals will be able to standardise terminology and have a common language, contributing to the impact of having a taxonomy in European Portuguese.

## Data Availability

The dataset and additional material are available at Open Science Framework repository (OSF), (https://doi.org/10.17605/OSF.IO/8HDJK) in Translating the Behaviour Change Technique Taxonomy v.1 into European Portuguese ( Félix *et al.*, 2022) This study contains the following underlying data: General guidance agreed for the independent forward translation ( https://osf.io/8hdjk/files/osfstorage/63dd19fd6946a0042d7a4412) Example of a feedback form. ( https://osf.io/8hdjk/files/osfstorage/63eaa675a3fade03ede7dedf) Independent forward translation originating from step 1 and the reconciled version of the BCTTv1-PT (step 2) ( https://osf.io/8hdjk/files/osfstorage/63dd2a907d01870475bc4cd4) Final version BCTTv1-PT ( https://osf.io/8hdjk/files/osfstorage/63ea8727eeb8ff03e22113e5) Data are available under the terms of the Creative Commons Attribution 4.0 International license (CC-BY 4.0).
