# Supplementary material for: Translating the Behaviour Change Technique Taxonomy v.1 to other languages: the approach used in European Portuguese (BCTTv1-PT)
Source: Wellcome Open Res. 2024 Mar 1;9:114. [Version 1] doi: 10.12688/wellcomeopenres.20609.1 (PMC11541075; doi:10.12688/wellcomeopenres.20609.1)
Supplement: Peer review history

# Reviewer report: Reviewer response for version 1

**DOI:** 10.21956/wellcomeopenres.22812.r106700

**Published:** 2024-11-06

**Authors:** Judith Dyson

**Affiliations:** 1 Department of Health, Education and Life Sciences, Birmingham City University, Birmingham, England, UK

The authors translated the BCT taxonomy v 1 to Portuguese following a robust and detailed, clearly documented process. My only suggestion for improvement might be some discussion on the cultural appropriateness on the BCTs which have been mostly used in similar cultures (UK, America, Canada, Australia). Europe is the most diverse continent and I would be interested to hear more about how culturally acceptable techniques are in future work. Michael Borg Prof IPC has used the TDF/BCT approach widely and has written about the challenges of application in Malta. I would recommend chatting with him as he is engaged in an innovative cross Europe IPC improvement project currently supporting translation of a barriers questionnaire for 14 different countries and with plans to select BCTs in the next two years.

Is the work clearly and accurately presented and does it cite the current literature?

Yes

If applicable, is the statistical analysis and its interpretation appropriate?

Yes

Are all the source data underlying the results available to ensure full reproducibility?

Yes

Is the study design appropriate and is the work technically sound?

Yes

Are the conclusions drawn adequately supported by the results?

Yes

Are sufficient details of methods and analysis provided to allow replication by others?

Yes

Reviewer Expertise:

Implementation science, psychology, improvement, behaviour change (clinical and health)

I confirm that I have read this submission and believe that I have an appropriate level of expertise to confirm that it is of an acceptable scientific standard.

# Reviewer report: Reviewer response for version 1

**DOI:** 10.21956/wellcomeopenres.22812.r88743

**Published:** 2024-08-07

**Authors:** Madalina Jäger, Kristina Pfeffer

**Affiliations:** 1 University of Southern Denmark, Odense, Denmark; 2 Department of Sports Science and Clinical Biomechanics(Ringgold ID: 535231), University of Southern Denmark, Odense, Region Syddanmark, Denmark

**Summary**

 This manuscript highlights the critical role of behavior change interventions (BCIs) and the need for accurate descriptions to improve their design, implementation, and evaluation. It also emphasizes the importance of using a standardized language to describe BCIs, facilitating systematic reviews, replication, and practical application. The focus of the report is to describe the approach to translating and adapting the BCTTv1 from English to European Portuguese. The methodology details the process of adapting the BCTTv1 into European Portuguese, using a structured approach. The translation process involved  a collaborative iterative approach involving four phases: independent forward translation, forward translation reconciliation,  expert consultation and feedback analysis.

 The successful adaptation of BCTTv1 into European Portuguese demonstrates the feasibility of translating complex taxonomies into different languages while maintaining  integrity and accuracy. This process can serve as a model for similar endeavors in other languages, ultimately contributing to the global standardization of behavior change interventions. ​​

 Both me and my co-reviewer  thoroughly enjoyed reviewing this manuscript and only have some minor requests for revision.

 The strengths of this work include: a detailed and transparent translation and adaptation process,  solid and rigorous translation methodology, the involvement of multiple experts to ensure accuracy and cultural relevance, the translation of BCTTv1 is innovative but also extending existing work.

 Considering these strengths, though, as I read the manuscript I found some areas in

 which I would have appreciated greater clarity and detail.

 Is the work clearly and accurately presented and does it cite the current literature?Partially

**Abstract**

*and were enacted **entirety-** Spelling (*replace with *entirely)*

**Background**

- *European Portuguese*- **Consider adding some information about the Portuguese language and countries where it is spoken. Also, consider adding a foot note explaining the difference between European Portuguese and Brazilian Portuguese.**
- *BCTTv1 applies to an extensive range of behaviour change Interventions-* **Would it be more correct to say – *BCTTv1 has been applied to a range of behaviour change interventions?***
- *This study reports a structured approach to adapt-* **change to *a structured approach to adapting*** Could a little more background regarding translation literature be included in the Introduction?
- Why did you choose to translate the BCTTV1 and not the new Behaviour Change Ontology?
- Are sufficient details of methods and analysis provided to allow replication by others?Partially

**Methods**

*The forward translation from English to Portuguese was carried out by two independent translators-* **How was the translation performed? Did the translators use any software/ AI ?**

*Experts disagreed with the translated definitions in 40.9% of the BCTs (38/93)* **There was a rather large number of changes needed. Can you clarify the reason (s) ?**

*(2) consistency among definitions of different BCTs; a term regarded as more common in European Portuguese.* **Perhaps the changes also reflect the choice between labels that have a similar meaning, but one of them is closer to the specific BCT (e.g. comprometimento and compromisso)**

*a more common term used Portuguese- used **in** Portuguese*

*Overall, recommendations intended mainly to guarantee harmonization across BCTs* *wording (e.g., changing the infinitive form of verbs to non-infinitive forms) or to adapt the*

*wording to the Portuguese context.* **Perhaps more examples would be useful**

**Results**

*Table 1- Recommendations **-*** **Very useful table with examples. It would have been even better if the reasoning behind the changes was specified as well.**

*Figure 2- BCT definitions- Is* **there any pattern that you observed- why did some groupings need more changes than others? e.g. shaping knowledge**

**Discussion**

*Our work has important implications for research, education and practice, both in Portugal and other Portuguese-speaking countries-* **Very good argument. I expected to read something like this already in the Background section.**

**Can you add some examples of groups who would benefit from this tool?**

*Interventions delivered by professionals in a national research context may benefit from using BCTTv1-PT to increase fidelity, as the original English version is prone to unintended deviations in meaning.* **Can you elaborate on how and why this would increase fidelity?**

*A limitation of this study is that the final version of BCTTv1-PT has not been pilot tested.*

**Could you specify why this version was not pilot tested? Perhaps it would be useful to mention something about what the process of pilot testing this version would entail.**

Is the work clearly and accurately presented and does it cite the current literature?

Partly

If applicable, is the statistical analysis and its interpretation appropriate?

Yes

Are all the source data underlying the results available to ensure full reproducibility?

Yes

Is the study design appropriate and is the work technically sound?

Yes

Are the conclusions drawn adequately supported by the results?

Yes

Are sufficient details of methods and analysis provided to allow replication by others?

Partly

Reviewer Expertise:

Developing and evaluating complex health interventions; Theories and models of behavior change, Self-management support; Mixed method research

We confirm that we have read this submission and believe that we have an appropriate level of expertise to confirm that it is of an acceptable scientific standard.
